# Investigating pre‐registration podiatry students approaches to identifying dermatology conditions in different skin tones: A mixed methods protocol

**DOI:** 10.1002/jfa2.70015

**Published:** 2024-11-30

**Authors:** Simon Otter, Deborah Whitham, Gianluca Melotto, Lauren Mann, Yaa Agyare, Joanne Gozo‐Reyes, Faye Funnell, Alex Sykes, Penny Dale

**Affiliations:** ^1^ School of Health & Rehabilitation Science Health Sciences University Bournemouth UK; ^2^ Centre for Regenerative Medicine and Devices University of Brighton Brighton UK; ^3^ School of Sport & Health Sciences University of Brighton Brighton UK

**Keywords:** decolonizing education, health inequalities, mixed methods, podiatry, skin tone diversity

## Abstract

**Background:**

Health inequalities are a well‐known and widespread phenomenon throughout health care settings. In particular, people of color experience higher rates of delayed and/or misdiagnosis contributing to poorer outcomes and an increased mortality risk. Research suggests that health care professionals find it more difficult to correctly diagnose dermatological conditions in the non‐White patient demographic. Although podiatrists routinely examine and assess skin lesions, there is a paucity of research exploring their accuracy or confidence in recognizing skin pathologies. This study aims to investigate podiatry student's ability, confidence, approaches, and perceptions in diagnosing dermatology pathologies in different skin tones.

A mixed methods exploratory sequential design is proposed. In stage one, podiatry students from different higher education institutions will be invited to complete a pictorial survey. We have designed a survey comprising six validated images of inflammatory skin pathology (either eczema or psoriasis) in three different skin tone categories, standardized using the Fitzpatrick scale. Data from the survey in stage one will then be utilized to inform the next stage of the research. In stage two, respondents who completed the initial survey will be invited to participate in focus groups to explore their perceptions surrounding diagnostic approaches, confidence, and perceptions of skin conditions in different skin tone. A process of thematic analysis will be employed to identify emergent themes from these data.

**Methods:**

A mixed methods exploratory sequential design is proposed. In stage one, podiatry students from different higher education institutions will be invited to complete a pictorial survey. We have designed a survey comprising six validated images of inflammatory skin pathology (either eczema or psoriasis) in three different skin tone categories, standardized using the Fitzpatrick scale. Data from the survey in stage one will then be utilized to inform the next stage of the research. In stage two, respondents who completed the initial survey will be invited to participate in focus groups to explore their perceptions surrounding diagnostic approaches, confidence, and perceptions of skin conditions in different skin tone. A process of thematic analysis will be employed to identify emergent themes from these data.

## 1 INTRODUCTION

Disparities in health care across ethnic and racial minorities have been extensively investigated, and the health inequalities associated with variations in skin color remain a well‐known phenomenon [1, 2, 3, 4, 5, 6]. Health care professionals find it more difficult to diagnose dermatological conditions in patients of a non‐White background, consequently people of color face a higher proportion of delayed or misdiagnosis [2, 5, 7]. This leads to poorer prognoses and in some cases decreased survival rates compared to their White counterparts [4, 8, 9]. For example, the detection of melanoma by general practitioners was significantly better in people with lighter skin [10]. Similarly, Fenton and colleagues [11] reported squamous cell carcinoma and atopic dermatitis were accurately diagnosed more frequently in people with lighter skin tone by medical students. However, reasons for these diagnostic differences are complex and multifactorial.

**FIGURE 1:**
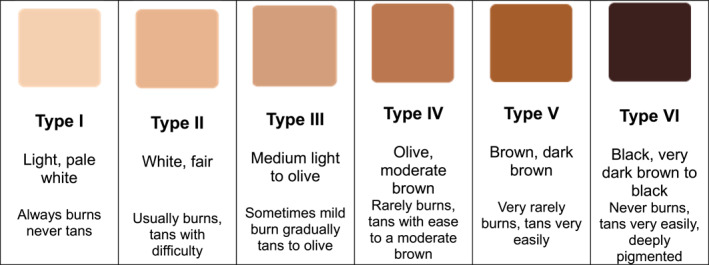
The Fitzpatrick skin phototype scale [14].

Skin phototype is determined by genetic background, ultraviolet light exposure, and chromophore distribution [12]. Skin of color is defined as a richly pigmented skin tone and typifying people from Asian, Hispanic, Native American, Native Hawaiian, Middle Eastern, Caribbean Black, African American, or African backgrounds who are classified as types 4–6 on the Fitzpatrick phototype scale [13] (Figure 1). Physiological differences between skin phototypes impact skin function in terms of the incidence and prevalence of certain dermatological conditions [15]. For instance, higher levels of melanin are considered protective against UV rays and consequently against certain skin cancers [16]. Therefore, the prevalence of common skin cancers is lower in people with darker skin phototypes [17], which may also explain some disparities in diagnosis rates. Skin pathologies present with significant differences across the spectrum of skin tone. Practitioners therefore need to be mindful of the fact that common dermatological disorders have specific clinical presentations in different skin phototypes [18]. For example, inflammatory skin lesions are typically characterized by redness in the lighter skin phototypes, and hyperpigmentation in darker skin types [15, 19, 20].

Turbes and colleagues [21] report medical textbooks consistently underrepresent patients with skin of color and/or from ethnic minorities risking a promotion of ‘whiteness’ as the norm. A study analyzing three of America's leading medical textbooks reported 4,146 images, in which 74.5% represented light skin tones, 21% medium skin tones, and only 4.5% dark skin tones [22]. Similarly, analysis of powerpoint slide decks in a North American medical school revealed 78.4% images were white and the remaining minority were from persons of color [23]. The lack of diversity and absence of dark skin tone images may mean that health care students are educated to detect, diagnose, and treat dermatological conditions primarily on the basis of light skin tones, despite the aforementioned differences in clinical presentation [19, 22]. The gap in knowledge and potential for a biased approach can lead to inaccuracies in diagnosis and a failure to detect serious conditions in a timely manner [24]. Consequently, preregistration education may, however inadvertently, embed health inequalities by limiting diagnostic confidence and ability in graduates.

Finally, online resources can be used by both patients and health professionals alike to research health conditions. Despite its popularity, a review of Google found only 5.7% of the images of dermatological conditions were in dark skin tones, whereas the majority of images were of white and light skin tones [25]. This lack of diverse online images depicting dark skin tones could further compound diagnostic error by health professionals. Kurtti et al. [25] further report the lack of representation in online images means people with skin of color may have a lower level of understanding of their own health and importantly when to seek help. Taken together, the overrepresentation of lighter skin tones means that the ability to recognize or diagnose and treat dermatological conditions in patients with skin of color can be impaired, adversely affecting the quality of care.

Podiatrists are key members of the health care team and routinely examine and treat dermatological conditions affecting patients' lower limbs and feet. The NHS long‐term plan highlights the need for confidence and competence in providing high quality and equitable care to patients of all backgrounds [26]. Moreover, the new delivery plan for recovering access to primary care [27] highlights the need for capacity building to enable patients to access the wider health care team. It is worth noting that 14.2% of the general practice consultations include skin lesions [28]. There are no easily identifiable published reports regarding the diagnostic accuracy of dermatological lesions among podiatrists, particularly in skin of different color. Recently, there has been a pedagogical imperative to ‘decolonize the curriculum’ seeking to end the over‐representation of a Eurocentric/Western epistemological lens in favor of a more diverse curricula [29, 30]. Improving wider representation aims, among other goals, to improve the experience and academic outcome of students from marginalized and under‐represented groups [31]. Integrating and enhancing these perspectives offers greater insight into culturally diverse populations and the health issues they face [32]. Substantial efforts have been made across the higher education sector to ‘decolonise’ curricula and pedagogy and most higher education institutes offer toolkits (such as the one from SOAS [33]) and/or inclusive curricula health‐checks (such as the example from ULC [34]). Current health care students are likely to have been exposed to this change in curricula in a way that many qualified practitioners might not [35]. Consequently, we sought to explore pre‐registration podiatry students' diagnostic approach to inflammatory skin lesions in different skin tones and identify potential barriers to confidently diagnose common dermatological conditions across different skin phototypes.

## 2 METHODS

### 2.1 Study design

A mixed‐methods exploratory sequential design is proposed utilizing an interpretivist approach to our analytical processes. Mixed methods research designs seek to integrate quantitative and qualitative approaches into one study to enhance understanding, uncover patterns, and offer different perspectives on the issues highlighted that neither approach could do alone [36, 37, 38, 39]. In our study a two‐stage approach is planned. Stage one will consist of a pictorial survey of images in different skin tones and participants select the correct diagnosis from a selection of different skin complaints. This information seeks to inform stage two, which will comprise a series of focus groups to enable a deeper exploration of decision‐making, confidence, limitations, and understanding. This study is designed in accordance with ASSESS principles for reporting mixed methods studies [40].

### 2.2 Ethical considerations and reflexivity

Ethical approval was granted by the University of Brighton, School of Sport and Health Science Research Ethics Committee (ref 2022–9784). Throughout the study strict confidentially and anonymity will be upheld. The research team was purposively selected to be inclusive of a range of individuals with different cultural and ethnic backgrounds to offer a wider epistemological standpoint. Equally, the researchers recognize that our position as podiatrists may influence our data analysis. To mitigate this potential bias, all researchers will complete online training associated with focus group management. In addition, methodologically, we will seek respondent validation throughout to ensure our interpretation of results is a true reflection of our participants' thoughts and perceptions.

### 2.3 Questionnaire design

Owing to a lack of validated instruments in the literature, a pictorial survey was initially developed de novo to assess podiatrists' diagnostic accuracy in different skin phototypes. A pictorial survey was preferred to support and encourage subsequent focus group discussions, explore the reasoning behind podiatrists' diagnoses, and enhance the likelihood of obtaining relevant data during the qualitative phase of the study, utilizing a process similar photo elicitation [41]. For the pictorial survey, the Fitzpatrick's classification [13] was used to standardize different skin phenotypes. A series of validated images of dermatological complaints were obtained (with consent) from the Primary Care Dermatology Society [42]. Limitations of suitable medical images for teaching and research is an established problem [22, 23, 43] and we found a paucity of suitable images available even from respected and trusted sources. Consequently, we were forced to collapse the six phototypes originally proposed by Fitzpatrick into three groups (Table 1).

**TABLE 1:** Researchers skin phototypes allocation based on the Fitzpatrick's phototypes classification [adapted from 14].

Researchers' skin phototypes allocation	Fitzpatrick phototypes	Fitzpatrick phototypes description
Light skin phototype group	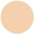 Fitzpatrick's phototype I	Always burns, never tans
	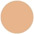 Fitzpatrick's phototype II	Usually burns, tans less than average (with difficulty
Medium skin phototype group	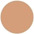 Fitzpatrick's phototype III	Sometimes mild burns, tans about average
	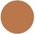 Fitzpatrick's phototype IV	Rarely burns, tans more than average (with ease)
Dark skin phototype group	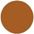 Fitzpatrick's phototype V	Rarely burns, tans deeply
	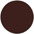 Fitzpatrick's phototype VI	Never burns, tans deeply

Table 1 shows how researchers combined the six Fitzpatrick's phototypes (central column) into three groups (left column): light skin phototype, medium skin phototype, and dark skin phototype. An image of psoriasis and eczema for each skin phototype group was retrieved to generate a six‐image pictorial survey. The right column provides an outline description of Fitzpatrick's phototypes.

Given the images available, we chose two inflammatory skin conditions, psoriasis and atopic eczema to assess podiatrists' diagnostic accuracy. Our rationale was two‐fold. Firstly, podiatrists are highly likely to see patients with either of these skin conditions, particularly given increased possibility of secondary complications in these complaints, for example, bacterial infection. Secondly, there are important differences in clinical presentation and incidence across populations with different skin phototypes [16]. For example, psoriasis in patients with darker skin phototype is characterized by more scaling and thicker plaques, greater body involvement, less noticeable erythema and increased risk of hyperpigmentation than patients with lighter skin [44, 45]. Similarly, atopic eczema in darker skinned populations presents with less distinguishable redness and erythema and shows purple‐brown skin rashes [46, 47]. One image of psoriasis and one image of atopic eczema for each skin phototype group in Table 1 will be used. We sought to select images on both the anterior and posterior surfaces of the lower limb to focus on the nature and character of the lesion, not simply its anatomical location.

The initial version of the survey was completed by eight qualified podiatrists from a mix of settings (NHS, private practice and higher education) via an online request. Each podiatrist was invited to complete the questionnaire and comment on face validity and feasibility, which led to minor changes in layout, wording, and the choice of diagnostic possibilities presented in each question. The final version of the completed questionnaire consisted of six different images of psoriasis or eczema with a choice of five different differential diagnoses to choose from (supplementary file 1). A Cronbach's alpha was subsequently computed to assess the test–retest reliability of the pictorial survey (0.941), indicating a high level of consistency.

### 2.4 Focus group development

Focus groups will be conducted to explore and discuss the responses from the pictorial survey. Focus groups can provide a richness of data by promoting interaction and discussion between participants [48, 49]. Initially, two qualified podiatrists who completed the pictorial survey worked with researchers to develop the focus group schedule. To encourage discussion, researchers (GM, YA, and JG) selected the images with the highest diagnostic accuracy in the initial pictorial survey. The discussion sought to analyze clinical reasoning behind podiatrists' diagnoses and consideration of participants' confidence in making diagnoses. Verbatim transcription of discussions and nonverbal communication notes were combined into a single “preliminary transcript” that acted as a resource to develop and refine the focus group schedule and prompts. Researchers utilized a process of thematic analysis to systematically explore the diagnostic approach of skin lesions in different skin phototypes and investigate their perceived confidence. To ensure the acceptability and appropriateness of questions, the draft focus group schedule was returned to podiatrists to seek their option prior to completion. The final version of agreed focus group questions and prompts are available in supplementary file 2. One key recommendation was for small focus groups (<8 participants) to encourage participant sharing, particularly around sensitive topics such as perceived confidence in diagnostic ability [50, 51].

### 2.5 Main study

#### 2.5.1 Subjects and settings

Purposive nonprobability sampling will be undertaken from different cohorts of final‐year podiatry students from different higher education institutions across south/central England. Cohorts will be from different academic years to avoid potential ‘cohort effect’ associated with external factors beyond our control. Potential participants will be invited via email from a university‐based administrator to avoid coercion by academic tutors or the research team. Participants may include both preregistration MSc and/or BSc cohorts who attend similar teaching sessions and complete a similar number of placement hours (inclusion/exclusion criteria are detailed in Table 2).

**TABLE 2:** Participant inclusion/exclusion criteria.

Criteria	
Included	Be a final year student on either a preregistration or undergraduate podiatry course Aged 18 years or over Competency in English language to understand instructions and give full consent to participate Similar levels of clinical practice and academic exposure to peers in that cohort
Excluded	Previous educational background/qualification in dermatology Students who have graduated from podiatry courses

#### 2.5.2 Questionnaire delivery

The pictorial questionnaire is designed to be completed in participants own setting on their own device over a 4‐week window to take account of formal academic commitments. To ensure anonymity and confidentiality, each participant will be allocated an individual identification number to use on the completion and return of the questionnaire to the research team. At the time of completion respondents will be asked if they would like to participate in a subsequent focus group.

#### 2.5.3 Focus group management

Following questionnaire completion and to maximize participation, focus groups will be held online via Microsoft Teams. Each focus group is intended to last approximately 60 min and are semi‐structured to prompt the contextualization and exploration of the questionnaire results with allowance for discussion of wider context and ideas. To support and augment the focus group, the correct answers to the questionnaire questions will be revealed, but without the revelation of the full study results. With consent, each focus group will be recorded using the Microsoft Teams recording facility and transcribed with the Teams live captions feature. Participants will have the option to have their cameras on or off and notes on social cues (head nodding/shaking, pauses, laughter, and changes in facial expression) will be noted, where possible, and added to the transcript in italics.

### 2.6 Data analysis

#### 2.6.1 Pictorial survey

Categorical data will indicate the total number of correct answers for each skin tone and the total number of correct respondents for each skin condition (either eczema or psoriasis). For ease of comparison all data will be presented as percentages.

#### 2.6.2 Focus group data

Prior to analysis, members of the research team will check transcripts against the audio recordings to confirm consistency and to gain familiarity with the data [52]. To ensure anonymity, all identifying factors in the transcripts will be removed and participants allocated a pseudonym. Each piece of extracted data will be analyzed, with key words and phrases highlighted in different colors for ease of differentiation, coded, and commented on with the purpose of searching for meanings and potential themes. Once the analysis process is complete, researchers will discuss and agree findings, organizing them into a set of themes once thematic saturation is achieved [52, 53]. Table 3 outlines the analysis process. Detailed coding and analysis of the data enables identification and refinement of complex themes and seeks to reveal a rich, thick textual description of the data that reflects the aims of the study. Once complete, themes will be returned to participants for respondent validation to enhance credibility and trustworthiness of the analysis process.

**TABLE 3:** Description of the processes undertaken by the researchers for thematic analysis [36, 52].

Stage of thematic analysis	Steps taken by the researchers
Familiarization with the data	Researchers individually corrected the automatic transcript of audio data by manual processes to ensure that data were transcribed verbatim. The researchers record their preliminary thoughts of the data during this process. Each researcher reads the transcript several times.
Coding	Researchers individually highlight and note specific and relevant features of the raw data to initiate the generation of codes. Researchers also note their own interpretation of the data where appropriate to ensure data are processed systematically and consistently.
Generating initial themes	Collectively the researchers searched the codes for patterns and themes. Firstly, color coding to group written codes and merge overlapping ones. Secondly, a digital thematic map of grouped codes to construct themes and link interpretations. The researchers ensured to possess flexibility throughout this process and discussion resolved any disagreements.
Reviewing themes	Researchers individually analyze the themes against the raw data to examine if they accurately reflect data. This process ensured there was no missing themes, and each theme was faithful to original data. Collectively the researchers record how they understood the themes and how the data supports them with the collating of verbatim transcript excerpts that accompany the associated theme. The researchers expand the thematic map with how the identified themes are directly relevant in answering the research question.
Defining and naming themes	Researchers collectively describe themes in detail with acknowledgment to the properties and specificity of the theme. All theme names are raw data excerpts (participant quotes) to ensure that the definitions are wholly reflective of the original data. The researchers define themes in alignment with the research aims and objectives and noted how the themes will reflect on the research outcomes.

### 2.7 Quantitative and qualitative component integration

Integration represented a multi‐level process, which aims to enhance value by increasing the strengths and reducing the weaknesses of the individual quantitative and the qualitative components [54, 55, 56]. In this study we adopted a contiguous approach where data integration took place at the design and method level as well as at the interpretation and reporting level [57]. Adopting an exploratory sequential design where one component informs the next represented the first level of integration between quantitative and qualitative components [58]. For the ease of understanding, quantitative and qualitative results are described separately but are intrinsically linked as the quantitative element informed the qualitative component of the study. Both elements are then combined in the analysis through the discussion.

## 3 DISCUSSION

The present study aims to investigate podiatrists' diagnostic accuracy and explore clinical reasoning for dermatological lesions seen in different skin phototypes, together with the potential barriers to current assessment techniques. Importantly, our design seeks to maximize the inherent advantages offered by differing methodological paradigms. The data collection period using different student cohorts seeks to overcome issues with smaller sample sizes that can limit generalizability of qualitative research. In addition, we chose students at different institutions to avoid any potential bias associated with a ‘cohort effect’.

Although diagnostic accuracy among other health professionals and practitioners has already been investigated for different dermatological conditions, [10, 12] to the best of our knowledge a mixed‐method study has never been adopted to further explore this type of clinical reasoning among podiatrists. The quantitative findings of this study were used to inform the qualitative phase, combining both approaches offered complementarity to reach an in‐depth understanding of a complex phenomenon [54]. For example, contrasting findings may be observed within the quantitative results as has been consistently demonstrated in studies among a wide range of health professionals [10, 11, 12, 59, 60].

Based on our reviews of the literature this research is the first mixed‐method study that seeks to investigate the dermatological diagnostic ability of podiatry students on different skin tones and therefore could be used as a starting point to explore this important topic further, both within and external to podiatric education. Our cross‐sectional design allowed educational variables to be controlled such as differences in teaching approaches and student prior knowledge, in so far as this is possible. The robust methodology and the retrieval of respondent validation increases the trustworthiness of our results [61]. Although our research design seeks to overcome potential limitations, we acknowledge some restrictions that are difficult to fully exclude in the design phase. The paucity of images in non‐White skin types offered an unexpected challenge to questionnaire design and highlights the on‐going need for this type of research. The lack of ‘real‐world’ cues such as anatomical location or medical history may offer a greater challenge for participants, but equally requires participants to focus on the characteristics of the lesion. Finally, our participants may have self‐selected based on a vested or predisposed interest in the topic and as such may limit generalizability. Nevertheless, identifying differences in diagnostic accuracy is an important first step to enhancing education to improving quality of care and working toward eliminating health disparities. The exploration of confidence whether grounded in aspects already identified in the literature; for example, the underrepresentation of darker skin phototypes in textbooks and/or exposure to demographic diversity, or for other yet unidentified reasons, offers an opportunity to enhance education practice both at pre‐registration and post qualification levels.

## AUTHOR CONTRIBUTIONS

**Simon Otter**: original draft (lead); conceptualization (supporting) supervision (equal). **Deborah Whitham**: Conceptualization (lead); review and editing (equal) Supervision (equal) **Gianluca Melotto**, **Lauren Mann**, **Yaa Agyare**, **Joanne Gozo‐Reyes**: methodology (equal) writing –formal analysis (equal), **Faye Funnell**, **Alex Sykes**, **Penny Dale**: writing –formal analysis (equal), review and editing (equal).

## CONFLICT OF INTEREST STATEMENT

The authors declare that they have no competing interests.

## ETHICS STATEMENT

Ethical approval was granted by the University of Brighton, School of Sport and Health Science Research Ethics Committee (ref 2022–9784). All participants provided informed, written consent.

## CONSENT FOR PUBLICATION

Not applicable.

## Supporting information

Supporting Information S1

## Data Availability

The datasets generated and/or analyzed during the current study are not publicly available due to participant confidentiality but are available from the corresponding author on reasonable request.

## References

[1] Riley, W. J. 2012. “Health Disparities: Gaps in Access, Quality and Affordability of Medical Care.” Transactions of the American Clinical and Climatological Association 123: 167–174. 23303983 PMC3540621

[2] Takeshita, Junko , Whitney T. Eriksen , Valerie T. Raziano , Claire Bocage , Lynn Hur , Ruchi V. Shah , Joel M. Gelfand , and Frances K. Barg . 2019. “Racial Differences in Perceptions of Psoriasis Therapies: Implications for Racial Disparities in Psoriasis Treatment.” Journal of Investigative Dermatology 139(8): 1672–1679: e1. 10.1016/j.jid.2018.12.032. 30738054 PMC6650313

[3] Gray, Alastair McIntosh . 1982. “Inequalities in Health. The Black Report: A Summary and Comment.” International Journal of Health Services 12(3): 349–380. 10.2190/XXMM-JMQU-2A7Y-HX1E. 7118327

[4] Charrow, Alexandra , Fan Di Xia , Cara Joyce , and Arash Mostaghimi . 2017. “Diversity in Dermatology Clinical Trials a Systematic Review.” Journal of the American Medical Association Dermatology 153(2): 193–198: PMID: 28055072. 10.1001/jamadermatol.2016.4129. 28055072

[5] Buster, Kesha J. , Erica I. Stevens , and Craig A. Elmets . 2012. “Dermatologic Health Disparities. 30.” Dermatologic Clinics 30(1): 53–59: viii. 10.1016/j.det.2011.08.002. 22117867 PMC3742002

[6] Egede, Leonard E. 2006. “Race, Ethnicity, Culture, and Disparities in Health Care.” Journal of General Internal Medicine 21(21): 667–669. 10.1111/j.1525-1497.2006.0512.x. 16808759 PMC1924616

[7] Oozageer Gunowa, Neesha , Marie Hutchinson , Joanne Brooke , Helen Aveyard , and Debra Jackson . 2021. “Pressure Injuries and Skin Tone Diversity in Undergraduate Nurse Education: Qualitative Perspectives from a Mixed Methods Study.” Journal of Advanced Nursing 77(11): 4511–4524. 10.1111/jan.14965. 34245169

[8] Yancy, Clyde W. 2020. “COVID‐19 and African Americans.” Journal of the American Medical Association 323(19): 1891–1892. 10.1001/jama.2020.6548. 32293639

[9] Marmot, Michael . 2020. “Health Equity in England: The Marmot Review 10 Years on.” British Medical Journal 368: m693. 10.1136/bmj.m693. 32094110

[10] Lyman, M. , Jo Mills , and Ar Shipman . 2017. “A Dermatological Questionnaire for General Practitioners in England with a Focus on Melanoma; Misdiagnosis in Black Patients Compared to White Patients.” Journal of the European Academy of Dermatology and Venereology 31(4): 625–628. 10.1111/jdv.13949. 27579938

[11] Fenton, Anne , Erika Elliott , Ashkan Shahbandi , Ekene Ezenwa , Chance Morris , Justin McLawhorn , James G. Jackson , Pamela Allen , and Andrea Murina . 2020. “Medical Students' Ability to Diagnose Common Dermatologic Conditions in Skin of Color.” Journal of the American Academy of Dermatology 83(3): 957–958. 10.1016/j.jaad.2019.12.078. 32017947 PMC7447081

[12] Taylor, Susan C. , A. Paul Kelly , Natalie E. Dupree , Alexa Boer Kimball , and Reva C. Lawrence . 2002. “Health Disparities in Arthritis and Musculoskeletal and Skin Diseases‐The Dermatology Session: National Institute of Arthritis and Musculoskeletal and Skin Diseases, 2000.” Journal of the American Academy of Dermatology 47(5): 770–773. 10.1067/mjd.2002.124691. 12399772

[13] Fitzpatrick, Thomas B. 1988. “The Validity and Practicality of Sun‐Reactive Skin Types I through Vi.” Archives of Dermatology 124(6): 869–871. 10.1001/archderm.1988.01670060015008. 3377516

[14] Fitzpatrick Skin Phototypes adapted from: https://www.arpansa.gov.au/sites/default/files/legacy/pubs/RadiationProtection/FitzpatrickSkinType.pdf

[15] Jothishankar, Balaji , and Sarah L. Stein . 2019. “Impact of Skin Color and Ethnicity.” Clinics in Dermatology 37(5): 418–429. 10.1016/j.clindermatol.2019.07.009. 31896399

[16] Abbas, Khizar , Muhammad Imran Qadir , and Sidra Anwar . 2019. “The Role of Melanin in Skin Cancer.” Critical Reviews in Eukaryotic Gene Expression 29(1): 17–24. 10.1615/CritRevEukaryotGeneExpr.2018024980. 31002590

[17] Cormier, Janice N. , Yan Xing , Meichun Ding , Jeffrey E. Lee , Paul F. Mansfield , Jeffrey E. Gershenwald , Merrick I. Ross , and Xianglin L. Du . 2006. “Ethnic Differences Among Patients with Cutaneous Melanoma.” Archives of Internal Medicine 166(17): 1907–1914. 10.1001/archinte.166.17.1907. 17000949

[18] Iwuala, C. , and S. C. Taylor . 2022. “Structural and Functional Differences in Skin of Colour.” Clinical and Experimental Dermatology 47(2): 247–250. 10.1111/ced.14892. 34388277

[19] Davis, S. A. , S. Narahari , S. R. Feldman , W. Huang , R. O. Pichardo‐Geisinger , and A. J. McMichael . 2012. “Top Dermatologic Conditions in Patients of Color: an Analysis of Nationally Representative Data.” Journal of Drugs in Dermatology 11(4): 466–473. 22453583

[20] Kundu, R. V. , and S. Patterson . 2013. “Dermatologic Conditions in Skin of Color: Part I.” Special Considerations for Common Skin Disorders 87(12): 850–856: American family physician. 23939567

[21] Turbes, Sandra , Erin Krebs , and Sara Axtell . 2002. “The Hidden Curriculum in Multicultural Medical Education.” Journal Association American Medical College 77(3): 209–216. 10.1097/00001888-200203000-00007. 11891157

[22] Louie, Patricia , and Rima Wilkes . 2018. “Representations of Race and Skin Tone in Medical Textbook Imagery.” Journal of Social Science and Medicine 202: 38–42. 10.1016/j.socscimed.2018.02.023. 29501717

[23] Martin, Glenna C. , Julianne Kirgis , Eric Sid , and Janice A. Sabin . 2016. “Equitable Imagery in the Preclinical Medical School Curriculum: Findings from One Medical School.” Academic Medicine 91(7): 1002–1006. 10.1097/acm.0000000000001105. 26839941

[24] Norman, Geoffrey R. , and Kevin W. Eva . 2010. “Diagnostic Error and Clinical Reasoning.” Medical Education 44(1): 94–100. 10.1111/j.1365-2923.2009.03507.x. 20078760

[25] Kurtti, Alana , Evan Austin , and Jared Jagdeo . 2022. “Representation of Skin Color in Dermatology‐Related Google Image Searches.” Journal of the American Academy of Dermatology 86(3): 705–708. 10.1016/j.jaad.2021.03.036. 33744357

[26] Stevens, S. 2019. NHS Long Term Plan [Internet]. NHS England: Available from: https://www.longtermplan.nhs.uk. Accessed May 9, 2023.

[27] NHS England . https://www.england.nhs.uk/publication/delivery‐plan‐for‐recovering‐access‐to‐primary‐care/. Accessed May 9, 2023.

[28] Le Roux, Emma , Peter J. Edwards , Emily Sanderson , Rebecca K. Barnes , and Matthew J. Ridd . 2020. “The Content and Conduct of GP Consultations for Dermatology Problems: a Cross‐Sectional Study.” British Journal of General Practice 70(699): e723–e730. 10.3399/bjgp20X712577. PMC7480176 32895240

[29] Begum, Neema , and Rima Saini . 2018. “Decolonising the Curriculum.” Political Studies Review 17(2): 1–6. 10.1177/1478929918808459.

[30] Abu Moghli, Mai , and Laila Kadiwal . 2021. “Decolonising the Curriculum beyond the Surge: Conceptualisation, Positionality and Conduct.” London Review of Education 19(1): 1–16. 10.14324/lre.19.1.23.

[31] Ahmed‐Landeryou, Musharrat . 2023. “Developing an Evidence‐Informed Decolonising Curriculum Wheel – A Reflective Piece.” Equity in Education and Society 2(2): 157–180. 10.1177/27526461231154014.

[32] Nazar, Mahdi , Kathleen Kendall , Lawrence Day , and Hamde Nazar . 2015. “Decolonising Medical Curricula through Diversity Education: Lessons from Students.” Medical Teacher 37(4): 85–393. 10.3109/0142159x.2014.947938. 25156358

[33] Decolonising the Curriculum Learning and Teaching Toolkit Available from: https://blogs.soas.ac.uk/decolonisingsoas/files/2018/10/Decolonising‐SOAS‐Learning‐and‐Teaching‐Toolkit‐AB.pdf last accessed 5.8.2024

[34] Inclusive Curriculum Health Check Available from.https://www.ucl.ac.uk/teaching‐learning/sites/teaching‐learning/files/ucl_inclusive_curriculum_healthcheck_2018.pdf last accessed 5.8.2024

[35] Dhoonmoon, L. , and J. Fletcher . 2022. “Assessing Skin Tones in Practice: Results of an International Survey.” Wounds International 13(2): 6–9.

[36] Johnson, R. Burke , and Anthony J. Onwuegbuzie . 2004. “Mixed Methods Research: A Research Paradigm Whose Time Has Come.” America Educational Research Association 33(7): 14–26. 10.3102/0013189x033007014.

[37] Cameron, R. 2011. “Mixed Methods Research: The Five Ps Framework.” Australian Institute of Business 9(2): 96–108.

[38] Shorten, Allison , and Joanna Smith . 2017. “Mixed Methods Research: Expanding the Evidence Base.” Evidence‐Based Nursing 20(3): 74–75. 10.1136/eb-2017-102699. 28615184

[39] Skamagki, Glykeria , Andrew King , Christine Carpenter , and Charlotte Wåhlin . 2022. “The Concept of Integration in Mixed Methods Research: a Step‐by‐step Guide Using an Example Study in Physiotherapy.” Physiotherapy: Theory and Practice 40(2): 1–8. 10.1080/09593985.2022.2120375. 36069530

[40] Ryan, Nessa , Dorice Vieira , Joyce Gyamfi , Temitope Ojo , Donna Shelley , Olugbenga Ogedegbe , Juliet Iwelunmor , and Emmanuel Peprah . 2022. “Development of the ASSESS Tool: a comprehenSive Tool to Support rEporting and Critical appraiSal of Qualitative, Quantitative, and Mixed Methods Implementation reSearch Outcomes.” Implement Science Communication 3(1): 34. 10.1186/s43058-021-00236-4. PMC8959802 35346390

[41] Hatten, K. , T. R. Forin , and R. Adams . 2013. “A Picture Elicits a Thousand Meanings: Photo Elicitation as a Method for Investigating Cross‐Disciplinary Identity Development.” 2013 ASEE Annual Conference and Exposition 23.89.1– 23.89.21. ISSN 2153‐5965.

[42] The Primary Care Dermatology Society ‐ UK. 2022.https://www.pcds.org.uk/. Accessed 9 May 2022.

[43] Alvarado, Savannah M. , and Hao Feng . 2021. “Representation of Dark Skin Images of Common Dermatologic Conditions in Educational Resources: a Cross‐Sectional Analysis.” Journal of the American Academy of Dermatology 84(5): 1427–1431. 10.1016/j.jaad.2020.06.041. 32565205

[44] Kerr, Gail S. , Seema Qaiyumi , John Richards , Hashem Vahabzadeh‐Monshie , Chesahna Kindred , Sean Whelton , and Florina Constantinescu . 2015. “Psoriasis and Psoriatic Arthritis in African‐American Patients‐‐the Need to Measure Disease Burden.” Clinical Rheumatology 34(10): 1753–1759. 10.1007/s10067-014-2763-337. 25164561

[45] Green, Liz . 2011. “An Overview and Update of Psoriasis.” Nursing Standard 25(35): 47–55. 10.7748/ns2011.05.25.35.47.c8498. 21667856

[46] Myers, Joan . 2015. “Challenges of Identifying Eczema in Darkly Pigmented Skin.” Nursing Children and Young People 27(6): 24–28. 10.7748/ncyp.27.6.24.e571. 26156613

[47] Manning, J. 2004. “The Assessment of Dark Skin and Dermatological Disorders.” Nursing Times 100(22): 48–51. 15195546

[48] Austin, Zubin , and Jane Sutton . 2014. “Qualitative Research: Getting Started.” Canadian Journal of Hospital Pharmacy 67(6): 436–440. 10.4212/cjhp.v67i6.1406. 25548401 PMC4275140

[49] Gundumogula, Manju . 2020. “Importance of Focus Groups in Qualitative Research.” International Journal Human Social Studies 8(11): 299–302. 10.24940/theijhss/2020/v8/i11/hs2011-082.

[50] Morgan, David L. 1996. “Focus Groups.” Annual Review of Sociology 22(1): 129–152. 10.1146/annurev.soc.22.1.129.

[51] Friedman, Daniela B. , Caroline Foster , Caroline D. Bergeron , Andrea Tanner , and S.‐Hill Kim . 2014. “A Qualitative Study of Recruitment Barriers, Motivators, and Community‐Based Strategies for Increasing Clinical Trials Participation Among Rural and Urban Populations.” American Journal of Health Promotion 29(5): 332–338. 10.4278/ajhp.130514-qual-247. 24670073

[52] Braun, Virginia , and Victoria Clarke . 2006. “Using Thematic Analysis in Psychology.” Qualitative Research in Psychology 3(2): 77–101. 10.1191/1478088706qp063oa.

[53] Lowe, Andrew , Anthony C. Norris , A. Jane Farris , and Duncan R. Babbage . 2018. “Quantifying Thematic Saturation in Qualitative Data Analysis.” Field Methods 30(3): 191–207. 10.1177/1525822x17749386.

[54] Halcomb, Elizabeth , and Louise Hickman . 2015. “Mixed Methods Research.” Nursing Standard: Promoting Excellence in Nursing Care 29(32): 41–47. 10.7748/ns.29.32.41.e8858. 25850508

[55] Small, Mario Luis . 2011. “How to Conduct a Mixed Methods Study: Recent Trends in a Rapidly Growing Literature.” Annual Review of Sociology 37(1): 57–86. 10.1146/annurev.soc.012809.102657.

[56] O’Cathai, A. , E. Murphy , and J. Nicholl . 2010. “Three Techniques for Integrating Data in Mixed Methods Studies.” British Medical Journal 341: c4587. 10.1136/bmj.c4587. 20851841

[57] Fetters, Michael D. , Leslie A. Curry , and John W. Creswell . 2013. “Achieving Integration in Mixed Methods Designs—Principles and Practices.” Health Services Research 48(6ii): 2134–2156. 10.1111/1475-6773.12117. 24279835 PMC4097839

[58] Zhang, Wanqing , and John Creswell . 2013. “The Use of “Mixing” Procedure of Mixed Methods in Health Services Research.” Medical Care 51(8): e51–e57. 10.1097/mlr.0b013e31824642fd. 23860333

[59] Sellheyer, Klaus , and Wilma F. Bergfeld . 2005. “A Retrospective Biopsy Study of the Clinical Diagnostic Accuracy of Common Skin Diseases by Different Specialties Compared with Dermatology.” Journal of the American Academy of Dermatology 52(5): 823–830. 10.1016/j.jaad.2004.11.072. 15858472

[60] Chisini, Luiz Alexandre , Thaís Gioda Noronha , Ezequiel Caruccio Ramos , Reginaldo Batista dos Santos‐Junior , Kaio Heide Sampaio , André Luis Faria‐e‐Silva , and Marcos Britto Corrêa . 2019. “Does the Skin Color of Patients Influence the Treatment Decision‐Making of Dentists? A Randomized Questionnaire‐Based Study.” Clinical Oral Investigations 23(3): 1023–1030. 10.1007/s00784-018-2526-7. 29934799

[61] Plummer, Prudence . 2017. “Focus Group Methodology. Part 2: Considerations for Analysis.” International Journal of Therapy and Rehabilitation 24(8): 345–351. 10.12968/ijtr.2017.24.8.345.

