# Supplementary material for: Investigating pre‐registration podiatry students approaches to identifying dermatology conditions in different skin tones: A mixed methods protocol
Source: J Foot Ankle Res. 2024 Nov 30;17(4):e70015. doi: 10.1002/jfa2.70015 (PMC11607545; doi:10.1002/jfa2.70015)
Supplement: Supplementary file 1 — Supporting Information S1 [file JFA2-17-e70015-s001.docx]

**Supplementary file 1**

PICTORIAL SURVEY

Instructions

The present questionnaire will show you six images of skin lesions. For each image, six diagnoses will be proposed (please, see the example below).

Skin lesion image

Option a

Option b

Option c

Option d

Option e

Option f

You are asked to tick and/or highlight the option that you think reflect the correct diagnosis. Only one answer is accepted (please, see the example below).

Skin lesion image

Option a

Option b

Option c


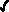


Option d

Option e

Option f

In case of multiple answers for a single image, the entire questionnaire will be excluded from the study.

THE PICTORIAL SURVEY WILL START ON THE NEXT PAGE

| Image 1: What is the most likely diagnosis for the skin lesion represented in this image? Please tick or highlight only one of the following options: |
| --- |
| 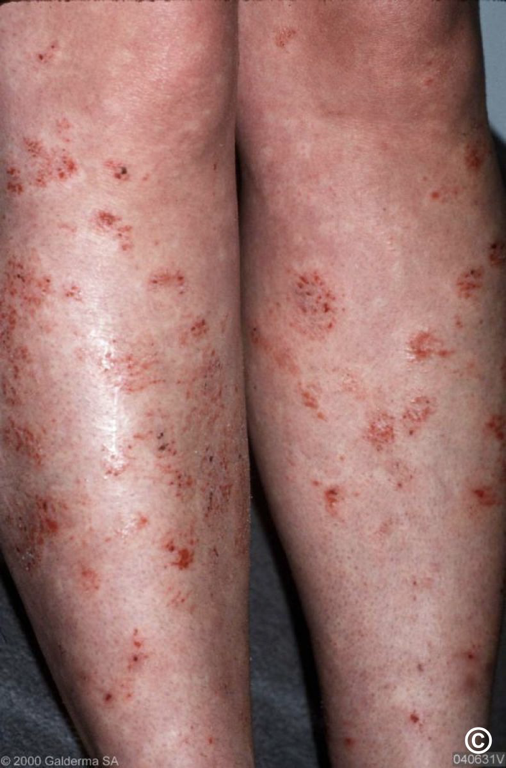 |
| Cellulitis  Psoriasis  Erythema nodosum  Scabies rash  Eczema  None of the above |

| Image 2: What is the most likely diagnosis for the skin lesion represented in this image? Please tick or highlight only one of the following options: |
| --- |
| 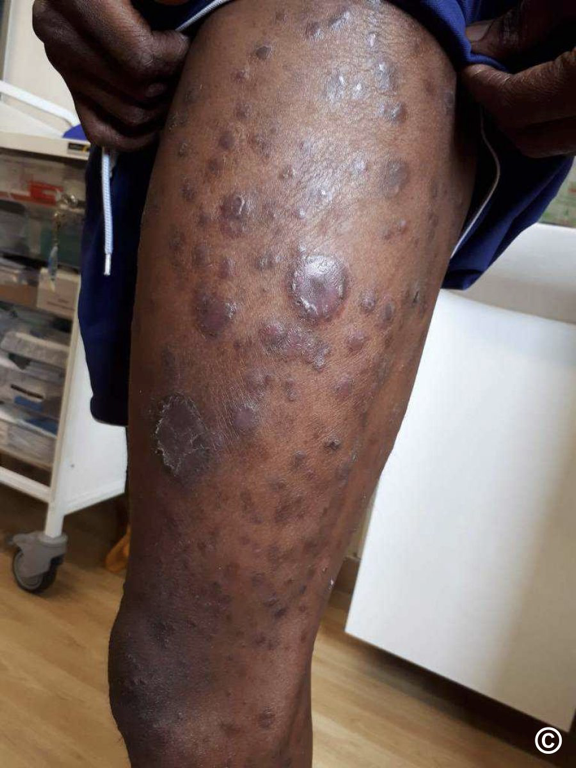 |
| Cellulitis  Psoriasis  Erythema nodosum  Scabies rash  Eczema  None of the above |

| Image 3: What is the most likely diagnosis for the skin lesion represented in this image? Please tick or highlight only one of the following options: |
| --- |
| 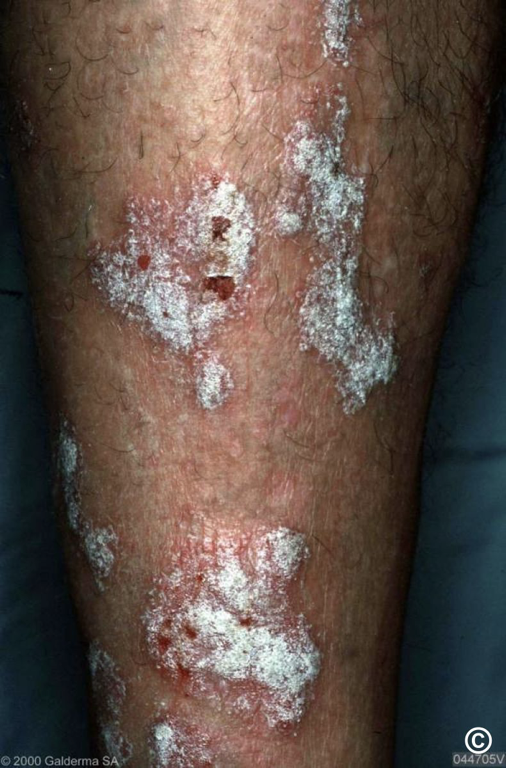 |
| Cellulitis  Psoriasis  Erythema nodosum  Scabies rash  Eczema  None of the above |

| Image 4: What is the most likely diagnosis for the skin lesion represented in this image? Please tick or highlight only one of the following options: |
| --- |
| 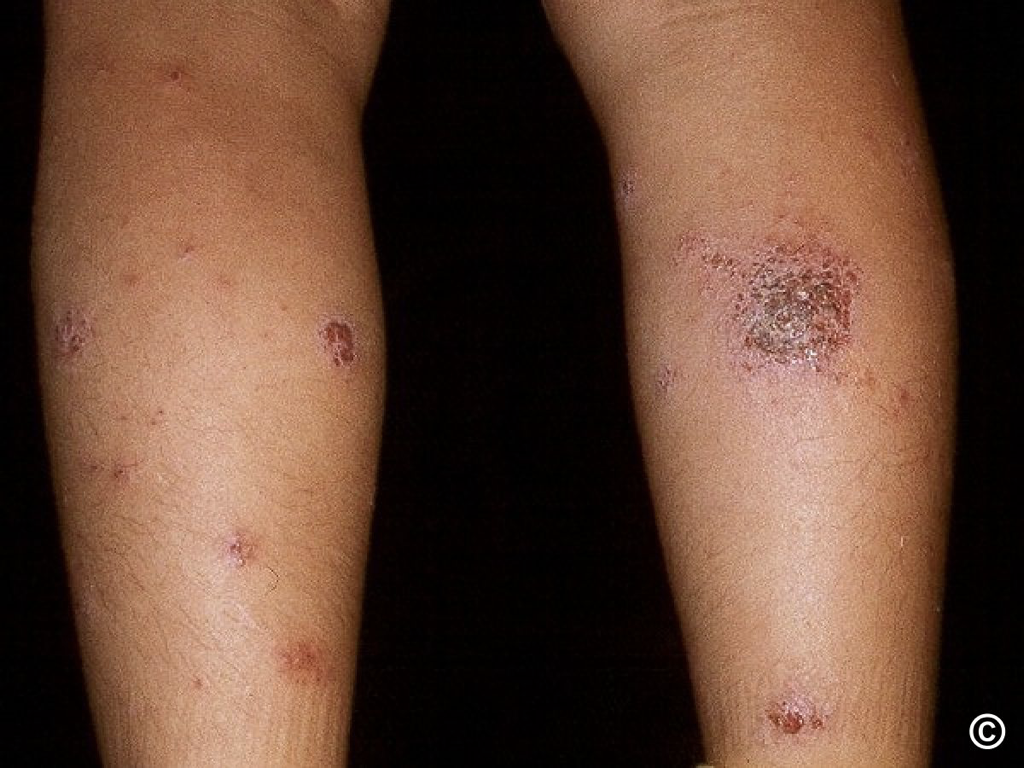 |
| Cellulitis  Psoriasis  Erythema nodosum  Scabies rash  Eczema  None of the above |

| Image 5: What is the most likely diagnosis for the skin lesion represented in this image? Please tick or highlight only one of the following options: |
| --- |
| 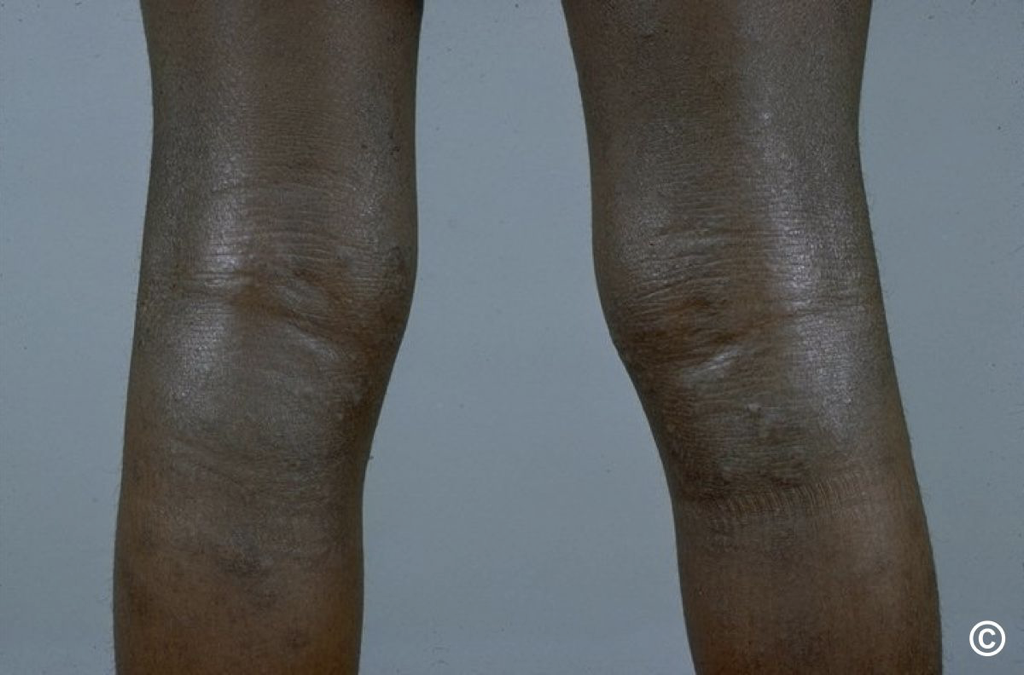 |
| Cellulitis  Psoriasis  Erythema nodosum  Scabies rash  Eczema  None of the above |

| Image 6: What is the most likely diagnosis for the skin lesion represented in this image? Please tick or highlight only one of the following options: |
| --- |
| 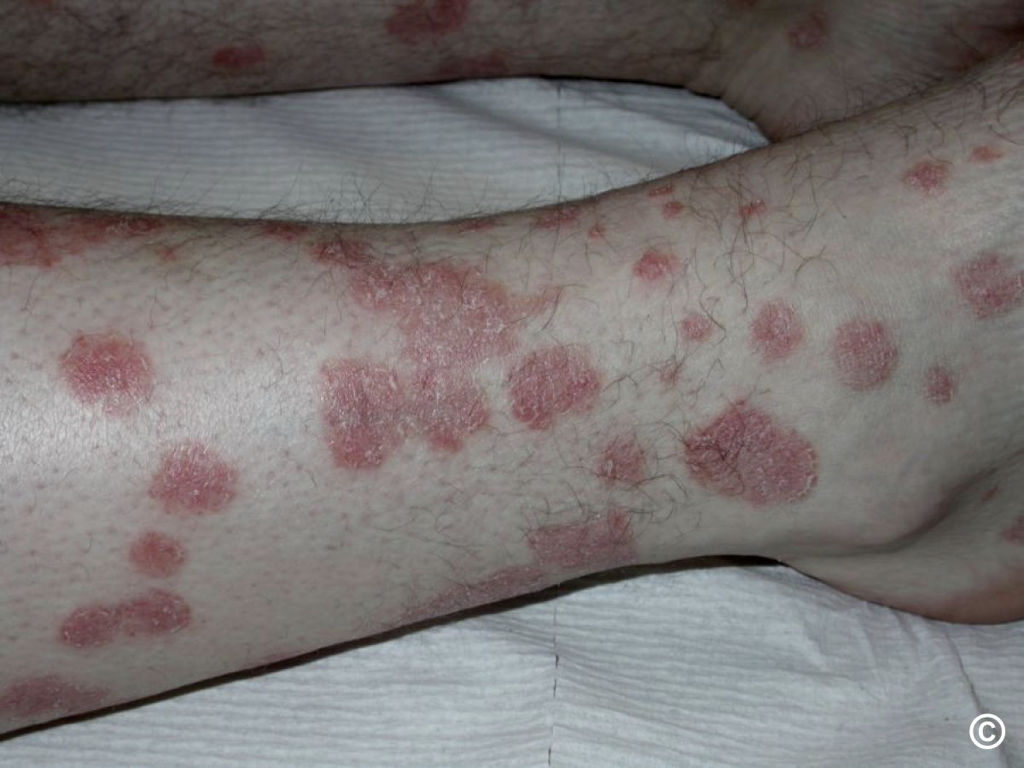 |
| Cellulitis  Psoriasis  Erythema nodosum  Scabies rash  Eczema  None of the above  The pictorial survey is over.  Please save and rename this document as per your identification number and email it back to (Name and Email)  Should you wish to participate in the focus group to explore this topic further, please indicate your availability in the same email.  The focus groups will be conducted online via Microsoft Teams (more details will be provided via email).  We are grateful for your time and availability in the participation of our study.  Thank you |

**Supplementary file 2.** Semi-structured interview questions for the focus groups

| Time expected (mins) | Description | Questions and prompts (if required) |
| --- | --- | --- |
| Welcome  0-5 | Welcoming participants and introducing research team  Reminding them we will be recording & prompting to turn on camera if they wish to do so, mention anonymity and confidentiality of the data  Ask participants to mute themselves when they are not speaking  Review the project aim | Terms for consistency  “As in the Participant information sheet your name will be unidentifiable to your input and the transcript will be anonymised and stored on the university OneDrive”  “The aim of this project is to investigate diagnostic approaches to dermatology conditions in podiatry education in different skin tones” |
| START RECORDING | | |
| Diagnostic Approach  5-30 | Researcher will show images in the chat box in the order of the questionnaire  Reveal correct diagnoses | For each image the question will be: “How did you approach diagnosis/ identifying clinical signs?”  Prompt: visual descriptors e.g. colour, size, shape, location, texture |
| Confidence in diagnosis and educational factors  30-55 | Firstly, the researchers will investigate the level of confidence in making diagnoses  Secondly the researchers will investigate the educational and/or social, clinical factors associated with the level of confidence in making diagnoses | Question  “How confident did you feel in diagnosing and detecting clinical signs across the different skin tones?”  Prompt:  “What makes you unsure/sure?”  Question  “How do you feel education/training contributes to your confidence in detecting clinical signs/ making diagnoses across the different skin tones?”  Prompt: barriers to representation and health equalities, dermatology teaching improves confidence  Question  “What has made you feel confident/ what could improve your level of confidence in detecting clinical signs/ making diagnoses in different skin tones”  Prompt: curriculum, patient demographics and exposure, classroom resources, teaching skills/aids, what could the university do in preparation?  Question  “What factors do you think podiatrists take into account when making dermatological diagnoses?”  Prompt: diagnostic language/ expectations used for dermatology e.g. red, erythema, rubor, pallor  CLOSING QUESTION: “Have we missed anything you would like to discuss/comment?” |
| STOP RECORDING | | |
| Conclusion and closing remarks  55-60 | Thank the participants  Any questions about the project |  |
